# Deep Learning-Based Method to Differentiate Neuromyelitis Optica Spectrum Disorder From Multiple Sclerosis

**DOI:** 10.3389/fneur.2020.599042

**Published:** 2020-11-30

**Authors:** Hyunjin Kim, Youngin Lee, Yong-Hwan Kim, Young-Min Lim, Ji Sung Lee, Jincheol Woo, Su-Kyeong Jang, Yeo Jin Oh, Hye Weon Kim, Eun-Jae Lee, Dong-Wha Kang, Kwang-Kuk Kim

**Affiliations:** ^1^Department of Neurology, Asan Medical Center, University of Ulsan College of Medicine, Seoul, South Korea; ^2^Asan Institute for Life Sciences, Asan Medical Center, Seoul, South Korea; ^3^Department of Medical Science, Asan Medical Institute of Convergence Science and Technology, Asan Medical Center, University of Ulsan College of Medicine, Seoul, South Korea; ^4^Clinical Research Center, Asan Institute for Life Sciences, Asan Medical Center, University of Ulsan College of Medicine, Seoul, South Korea; ^5^Department of Clinical Epidemiology and Biostatistics, Asan Medical Center, University of Ulsan College of Medicine, Seoul, South Korea

**Keywords:** brain magnetic resonance image (MRI), convolutional neural network (CNN), deep learning, multiple sclerosis, neuromyelitis optica spectrum disorder

## Abstract

**Background:** Differentiating neuromyelitis optica spectrum disorder (NMOSD) from multiple sclerosis (MS) is crucial in the field of diagnostics because, despite their similarities, the treatments for these two diseases are substantially different, and disease-modifying treatments for MS can worsen NMOSD. As brain magnetic resonance imaging (MRI) is an important tool to distinguish the two diseases, extensive research has been conducted to identify the defining characteristics of MRI images corresponding to these two diseases. However, the application of such research in clinical practice is still limited. In this study, we investigate the applicability of a deep learning-based algorithm for differentiating NMOSD from MS.

**Methods:** In this study, we included 338 participants (213 patients with MS, 125 patients with NMOSD) who visited the Asan medical center between February 2009 and February 2020. A 3D convolutional neural network, which is a deep learning-based algorithm, was trained using fluid-attenuated inversion recovery images and clinical information of the participants. The performance of the final model in differentiating NMOSD from MS was evaluated and compared with that of two neurologists.

**Results:** The deep learning-based model exhibited an area under the receiver operating characteristic curve of 0.82 (95% CI, 0.75–0.89). It differentiated NMOSD from MS with an accuracy of 71.1% (sensitivity = 87.8%, specificity = 61.6%), which is comparable to that exhibited by the neurologists. The intra-rater reliability of the two neurologists was moderate (κ = 0.47, 0.50), which was in contrast with the consistent classification of the deep learning-based model.

**Conclusion:** The proposed model was verified to be capable of differentiating NMOSD from MS with accuracy comparable to that of neurologists, exhibiting the advantage of consistent classification. As a result, it can aid differential diagnosis between two important central nervous system inflammatory diseases in clinical practice.

## Introduction

Neuromyelitis optica spectrum disorder (NMOSD) and multiple sclerosis (MS) are both inflammatory diseases of the central nervous system (CNS) (1). Because of the clinical and radiological similarities between the two, there has been a persistent debate on whether they are actually different. However, since the discovery of the anti-aquaporin-4 antibody (AQP4-Ab), which is an NMOSD-specific autoantibody (2), studies have confirmed MS and NMOSD to be distinct disease entities (3). Differentiating NMOSD from MS is of considerable importance in the field of diagnostics because the treatments for the two diseases differ considerably from each other, and disease-modifying therapies for MS can worsen NMOSD (4–6). Even though the presence of AQP4-Ab is essential for the diagnosis of AQP4-Ab-seropositive NMOSD, clinical and radiological differentiation between AQP4-Ab-seropositive NMOSD and MS remains crucial for the following reasons: (i) clinicians need to identify patients on whom the AQP4-Ab test should be performed; (ii) the result of an AQP4-Ab assay can be influenced by assay methodology and the patient's clinical status (3, 7, 8). Therefore, the role of brain MRI, which is the most common test for CNS inflammatory diseases, is significant in the differentiation between the two diseases (9).

Recently, machine learning-based algorithms have been applied to classify MRIs of patients with various neurological diseases (10–12). In particular, researchers have attempted to use various such methods, including multimodal data fusion and random forests, to differentiate NMOSD from MS (13, 14). Deep learning, which is a type of machine learning, does not require the specification of explicit features by experts and deduces most predictive features directly based on images (15, 16). Deep learning has been applied for classification based on medical images such as chest X-rays and fundus photographs, and it has been reported to exhibit excellent differentiation performance (17, 18). Fluid-attenuated inversion recovery (FLAIR) is an MRI technique that highlights T2 hyperintense lesions while suppressing cerebrospinal fluid (CSF) signals, thereby clearly revealing lesions that are in proximity to CSF, such as juxtacortical and periventricular lesions (19). FLAIR is considered to be superior to T2-weighted images in the context of the detection of MS brain lesions (20).

In this study, we aim to develop a deep learning model based on brain FLAIR MRIs and elementary clinical information to differentiate NMOSD from MS. Further, we evaluated the clinical applicability of the proposed model by comparing its performance with that of two neurologists.

## Materials and Methods

### Participants

We retrospectively reviewed the medical records of patients with MS and NMOSD who visited the Asan Medical Center, Seoul, Korea, between February 2009 and February 2020. Patients with MS who fulfilled the 2010 McDonald criteria and those with NMOSD with AQP4 immunoglobulin G (AQP4-IgG) seropositivity, in accordance with the 2015 International Consensus of NMOSD, were included in this study. The AQP4-IgG-seropositive status was confirmed via a commercial fixed cell-based assay (Euroimmun, Lubeck, Germany). Patients without available brain MRI data were excluded from the study. As a representative MRI of each patient, the last MRI was examined to use the latest data. All images were deidentified prior to being transferred to the study investigators.

### MRI Acquisition

2D FLAIR sequences were acquired using 1.5 T or 3.0 T scanners at the Asan Medical Center or other centers that referred patients and transferred images to the Asan Medical Center. The MRI protocol at the Asan Medical center is a fast spin-echo sequence with inversion recovery with the following scanning parameters: field of view = 220 × 200 mm; voxel size = 0.65 mm × 0.82 mm; a 336 × 231 acquisition matrix; time of repetition = 11,000 ms; time of echo = 125 ms; inversion time = 2,500 ms; slice thickness = 5 mm; slice gap = 2 mm; and number of acquisitions = 2.

### NMOSD and MS Classification by Neurologists

Two board-certified neurologists (H.W. Kim and Y.J. Oh) who have completed a 1-year clinical fellowship in neuro-immunology participated in this study as the human raters. They binarily diagnosed MS and NMOSD independently of each other by reviewing the FLAIR images alongside the following clinical information of each patient: age at disease onset, age at the time of MRI, sex, disease duration, and duration from last relapse. We considered these five variables to be essential to adequately capture the elementary information required for differential diagnoses in clinical practice.

### NMOSD and MS Classification via Deep Learning-Based Model

**Figure 1:**
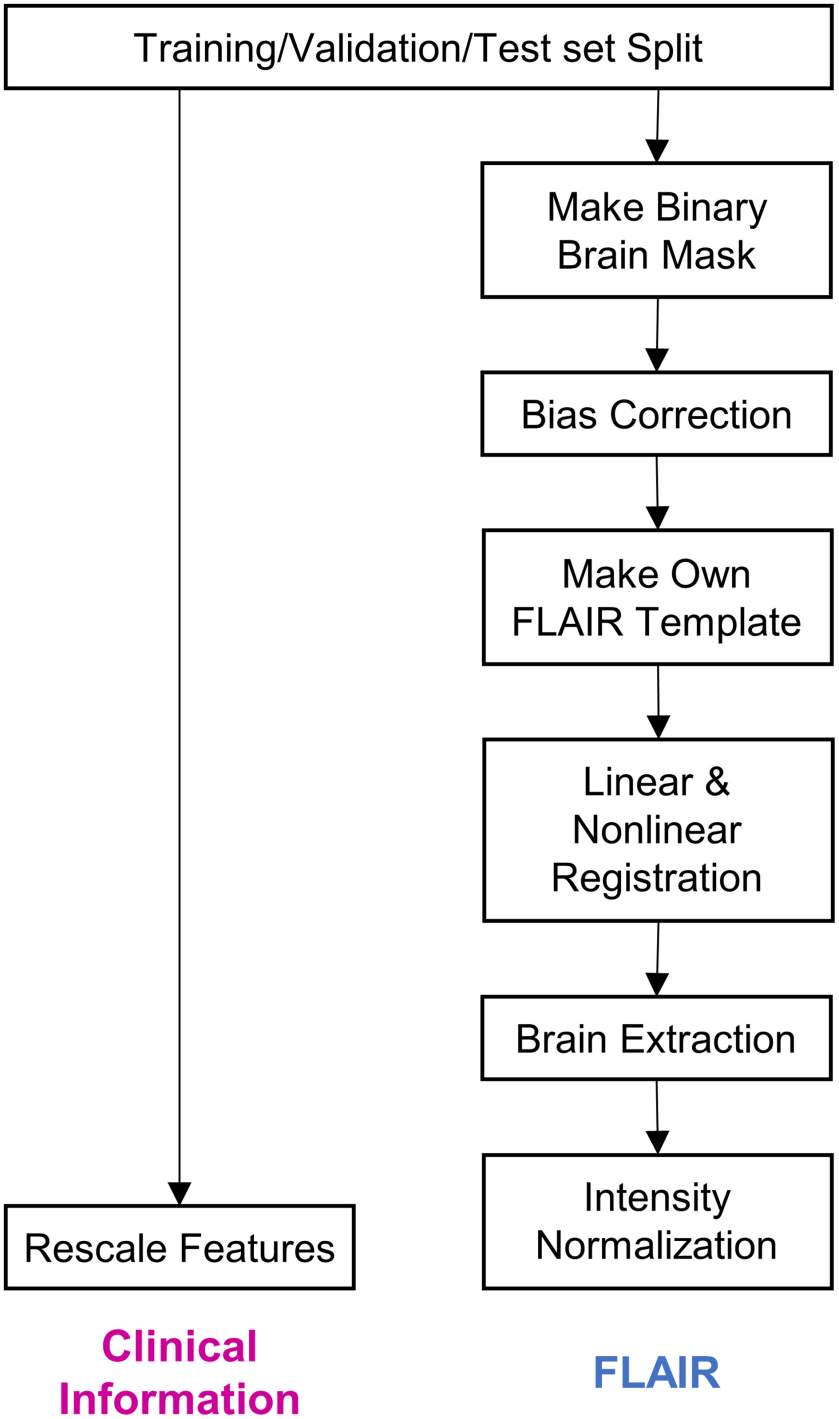
Steps of data preprocessing. The diagram depicts the preprocessing of data before it was input into the deep learning model. Firstly, the whole dataset was split into training, validation, and test sets. Subsequently, clinical information and fluid-attenuated inversion recovery (FLAIR) images were preprocessed separately. The details regarding the preprocessing procedure have been described in the Methods section.

All methods were implemented using Python and PyTorch for deep learning. The algorithm was executed on the Intel Core i7-8700K 3.70 GHz processor with two Nvidia GeForce RTX 2080 Ti graphics processing units. Figure 1 depicts the preprocessing steps whose details have been described below.

#### Subdivision of the Dataset Into Training, Validation, and Test Sets

We first split the dataset into training (45%, *n* = 152), validation (15%, *n* = 51), and test (40%, *n* = 135) datasets. We used stratified random sampling to ensure identical class ratios for every set.

#### Scaling Clinical Information

Four pieces of clinical information—age at disease onset, age at the time of MRI, disease duration, and duration from last relapse—were scaled to exhibit a median of 0 and an interquartile range of 1. As this scaling method uses statistics that are robust to outliers, the features can be transformed to exhibit nearly identical scales while being minimally affected by outliers.

#### Image Pre-processing

Brain mask data for each FLAIR sequence were obtained using a previously reported brain extraction algorithm (21). The brain mask data are presented as binary data, wherein only the voxels corresponding to brain parts are coded to correspond to 1. The FLAIR images were bias-corrected via the lesion prediction algorithm (22), as implemented in lesion segmentation toolbox (LST) version 2.0.15 (www.statistical-modeling.de/lst.html), for statistical parametric mapping (SPM, www.fil.ion.ucl.ac.uk/spm/). Following bias-correction, we obtained a file containing deformation parameters for each image; the parameters were capable of registering the image from its native space to the Montreal Neurological Institute (MNI) space with a resolution of 2 mm × 2 mm × 2 mm (23). Using these deformation parameters, we registered the MRIs belonging to the training set to the MNI space. Furthermore, using functional MRI of the brain software library (FSL) functions (24), these registered images were made to undergo 3D intensity normalization and were then averaged into one image, which was used as a template in this study. After creating the template, all the FLAIR images and brain masks were registered to the template and intensity normalization with Z-scoring was conducted.

#### Model Architecture

**Figure 2:**
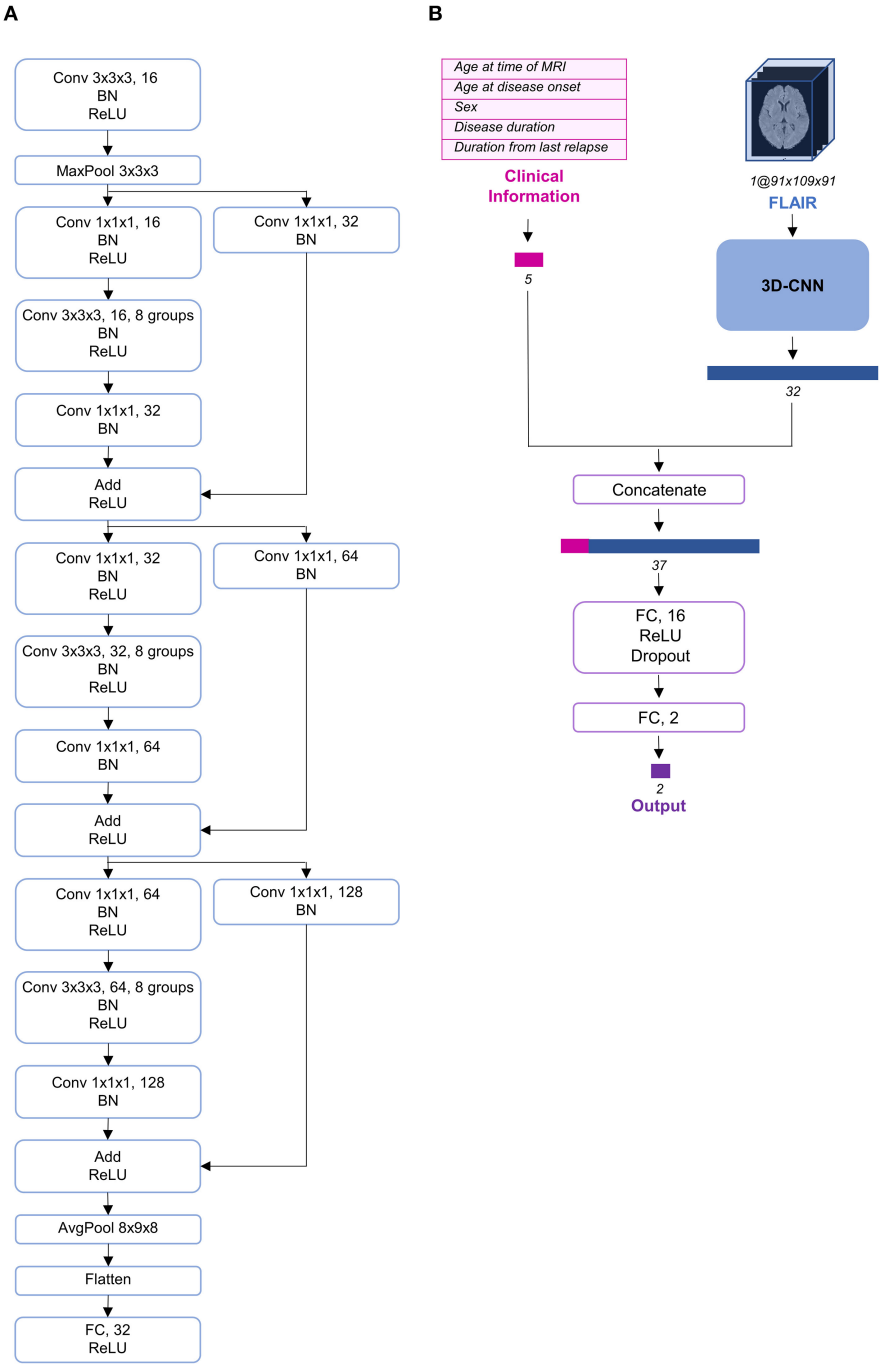
Schema of the model architecture. **(A)** The 3D convolutional neural network architecture used in our study. **(B)** The entire structure of the proposed model. The preprocessed fluid-attenuated inversion recovery (FLAIR) image was transmitted through the 3D convolutional neural network (CNN) and its feature vector was extracted and concatenated with the preprocessed clinical information. AvgPool, average-pooling; BN, batch normalization; Conv, convolution; FC, fully connected; MaxPool, max-pooling; ReLU, rectified linear unit.

Deep learning, particularly convolutional neural networks (CNNs), has been widely used in computer vision (25). Among the different variants of CNNs, ResNet has exhibited remarkable performance in image classification (26). Additionally, although most imaging studies have used 2D CNNs as their model architecture, some recent studies have proposed the use of 3D CNNs to fully utilize the spatial features of MRI and achieve better performance (27–30). Therefore, we constructed a 3D CNN architecture based on the idea of ResNeXt (31), which is a more developed model based on ResNet (Figure 2A). To use both clinical information and FLAIR data simultaneously, we concatenated the clinical information at the end of CNN architecture and propagated the information through two fully connected layers (Figure 2B).

#### Modeling

We trained and validated several models that differ from each other in terms of learning rate scheduling strategies, dropout rates, loss functions, and data augmentation strategies. After validating them using the validation set, we selected the best model. Stochastic gradient descent with a momentum of 0.9 and a weight decay of 10^−6^ was used as the optimizer. The initial learning rate of 0.025 was decayed using cosine annealing, and the pre-defined minimal learning rate, 0.015, was reached after 20 epochs. This cycle was repeated throughout the whole training process. This training strategy is called stochastic gradient descent with warm restarts (32). A batch size of 16 was selected and focal loss was used as a loss function for the best model, in which gamma and alpha were 1 and 0.25, respectively. Focal loss is often used when the class ratio of data is imbalanced (33). We expected the model to focus more on the NMOSD class by training it using focal loss. To prevent overfitting, rotation or translation was randomly applied to augment the FLAIR data immediately before they were input into the model. Further, the dropout strategy was used with a rate of 0.5. Both augmentation and dropout were conducted only during the training of the model and neither were applied during the validation or evaluation process. The final performance of the model was evaluated using the test set that had been split beforehand.

### Statistical Analysis

In this study, descriptive summaries were represented as frequencies and percentages for categorical variables and as mean ± standard deviation for continuous variables. In order to compare pairs of groups, Student's *t*-tests were used for continuous variables and Chi-squared tests were used for categorical variables. The diagnostic performances of the proposed model and the human raters (i.e., raters A and B) were measured in terms of sensitivity (i.e., correct ratio for the NMOSD group), specificity (correct ratio for the MS group), and accuracy (i.e., correct ratio regardless of the group) of their decisions related to the test set. Regarding sensitivity, specificity, and accuracy, a two-sided 95% exact confidence interval (CI) was calculated using the Clopper–Pearson method (34). McNemar's test was used to compare the decision performances of the deep learning-based model and the human raters. The diagnostic performance of the deep learning-based model was assessed using area under the receiver operating characteristic curve (AUC). The 95% CI of AUC was calculated following DeLong's method (35). Cohen's kappa (κ) test was adopted to evaluate the intra-rater reliability of the human raters between the first and second decisions on randomly duplicated 10% of identical samples in the test set; moreover, the aforementioned test was also performed to test the inter-rater agreement (on the test set) between the decisions of the two human raters and between the decisions of the human raters and those of the deep learning-based model. The strength of agreement based on κ was judged according to the following guidelines: <0.2 = slight; 0.2–0.4 = fair; 0.4–0.6 = moderate; 0.6–0.8 = substantial; and >0.8 = almost perfect (36). Two-sided *P*-values of < 0.05 were indicative of statistical significance. All statistical analyses were performed using SAS version 9.4 (SAS Institute Inc., Cary, NC).

## Results

### Baseline Characteristics of the Participants

We included 338 participants (213 patients with MS and 125 patients with NMOSD) in the study. The baseline characteristics of all participants have been presented in Table 1. The mean age at onset was lower for the MS group than that for the NMOSD group (33.1 ± 12.3 vs. 41.7 ± 13.7 years, *P* < 0.001). Fifty-five (25.8%) patients in the RRMS group and 13 (10.4%) in the NMOSD group were male. The mean disease duration and duration from last relapse were observed to be longer for the MS group. The percentages of MRIs performed at the Asan Medical Center (vs. other centers) and those of MRIs performed with a 3 T scanner (vs. 1.5 T scanner) were comparable between the two groups. Further, 30% (37/125) of brain MRIs from patients with NMOSD did not exhibit any T2 hyperintense lesions (≥3 mm). In contrast, all brain MRIs from patients with MS exhibited more than one T2 hyperintense lesion.

**Table 1:** Baseline characteristics.

	**MS**	**NMOSD**	***P*-value**
No.	213	125	
Male, *n* (%)	55 (25.8)	13 (10.4)	0.001
Age at onset, mean ± SD (years)	33.1 ± 12.3	41.7 ± 13.7	<0.001
Age at imaging, mean ± SD (years)	37.1 ± 12.0	45.9 ± 13.2	<0.001
Disease duration, mean ± SD (years)	7.6 ± 6.6	5.3 ± 5.6	0.001
Duration from last relapse, mean ± SD (years)	3.6 ± 4.3	1.2 ± 2.1	<0.001
EDSS score, mean ± SD	2.4 ± 1.8	3.3 ± 1.8	<0.001
MRI performed at AMC, *n* (%)	192 (90.1)	105 (83.3)	0.095
MRI performed with 3 T scanner, *n* (%)	172 (80.8)	91 (72.2)	0.089

*AMC, Asan medical center; EDSS, Expanded Disability Status Scale; NMOSD, neuromyelitis optica spectrum disorder; MS, multiple sclerosis; SD, standard deviation*.

### Diagnostic Performance

**Figure 3:**
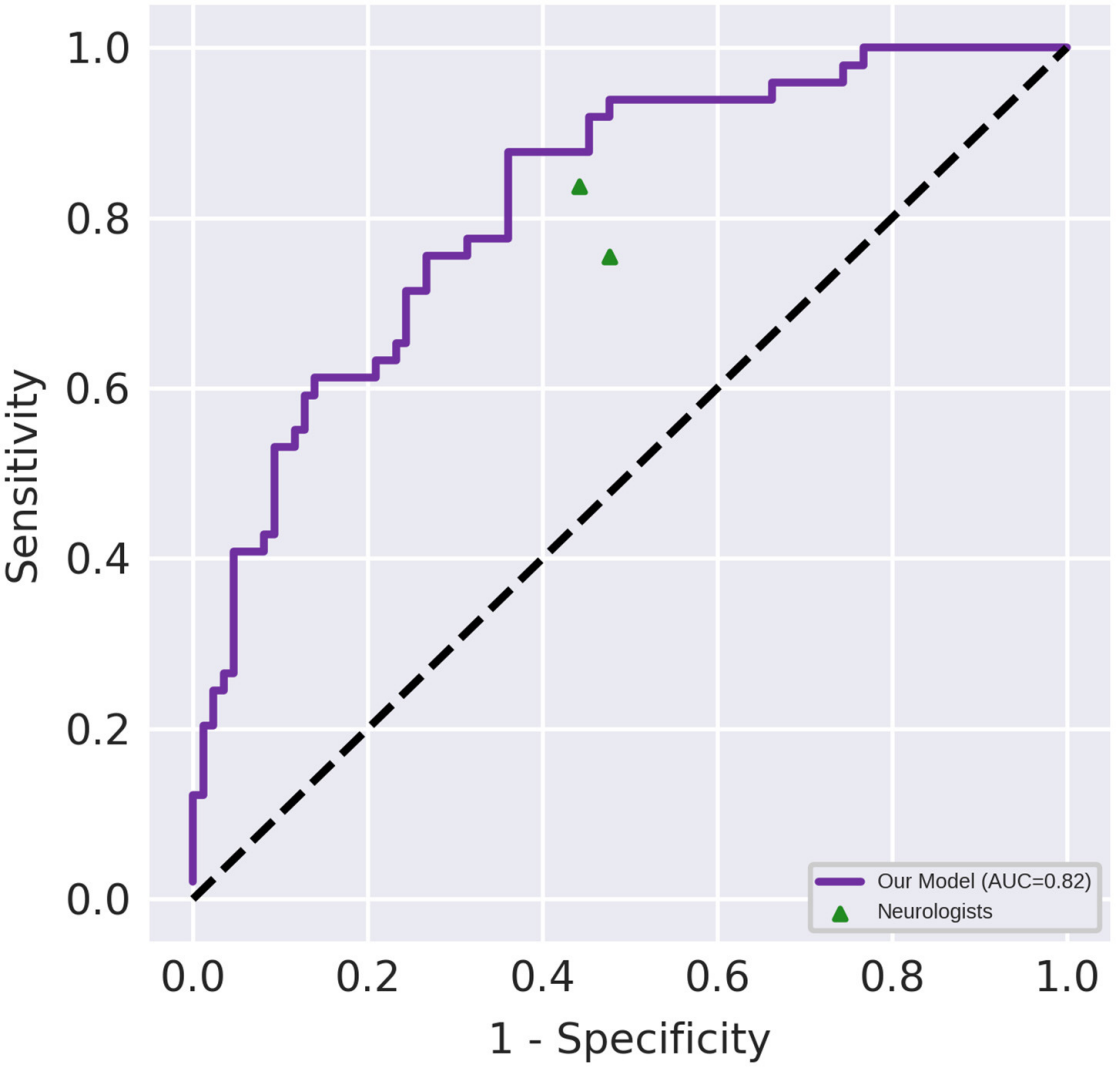
Diagnostic performance of the deep learning-based model and the neurologists. Performance on an independent test set with *n* = 135. The area under the receiver operating characteristic (AUC) of the proposed model (represented by the violet line) was 0.82 (95% CI, 0.75–0.89). The points correspond to the performance of the neurologists.

Figure 3 depicts the receiver operating characteristic curve of the model when it is evaluated using the test set (AUC, 0.82 [95% CI, 0.75–0.89]). The classification results of the human raters and deep learning-based model have been presented in Table 2. The accuracy of the deep learning model in differentiating NMOSD from MS was 71.1%, with a sensitivity of 87.8% and a specificity of 61.6%, all of which were comparable to those of the human raters. The intra-rater reliability of each human rater was moderate (κ of rater A = 0.471, κ of rater B = 0.500); the inter-rater agreement between the human raters was moderate (κ = 0.437); and the inter-rater agreements between the deep learning-based model and each human rater were fair and slight, respectively (κ = 0.288 between the deep learning-based model and rater A; κ = 0.154 between the deep learning-based model and rater B).

**Table 2:** Classification results.

	**Accuracy, %**	***P-*value**	**Sensitivity, %**	***P-*value**	**Specificity, %**	***P-*value**
Deep learning-based model	71.1 (62.7–78.6)	-	87.8 (75.2–95.4)	-	61.6 (50.5–71.9)	-
Rater A	65.9 (57.3–73.9)	0.382	83.7 (70.3–92.7)	0.754	55.8 (44.7–66.5)	0.511
Rater B	60.7 (52.0–69.0)	0.081	75.5 (61.1–86.7)	0.180	52.3 (41.3–63.2)	0.280

*Data have been presented with a 95% confidence interval. P-value for comparison with the deep learning-based model*.

## Discussion

In this study, we demonstrated that the proposed deep learning-based model is capable of differentiating NMOSD from MS with an accuracy of 71.1% (sensitivity = 87.8%, specificity = 61.6%), which was comparable to that achieved by the neurologists. Although the diagnostic accuracies were comparable, the deep learning-based model offers some advantages over human evaluation, including greater consistency in classification (intra-rater reliability κ = 1.0) and prompt reporting of results. The intra-rater reliability of the human raters was only moderate (κ = 0.47–0.50).

The definition of a brain lesion distribution as “at least one lesion adjacent to the body of the lateral ventricle and in the inferior temporal lobe or the presence of a subcortical U-fiber lesion or a Dawson's finger-type lesion” has been suggested as a criterion; this can be employed to distinguish patients with MS from those with NMOSD with 92% sensitivity and 96% specificity (37). In addition, the same criterion was used to distinguish MS from myelin oligodendrocyte glycoprotein antibody-associated diseases with a sensitivity of 90.9% and a specificity of 95.2% (38). However, the aforementioned studies did not include brain MRIs without T2 hyperintense lesions in their investigations, and the criterion included subjective definitions such as Dawson's fingers, which could be difficult to apply for clinicians who have little experience with CNS inflammatory diseases. In the current study, we included all MRIs with or without brain lesions and did not designate any specific lesion criteria. Instead, the deep learning-based model learned the most predictive features directly from the images. Therefore, we suggest that the proposed deep learning-based model is capable of overcoming the low practicability of the previously published brain lesion distribution criteria.

Two machine learning-based and one deep learning-based methods using brain MRI data had previously been developed to distinguish NMOSD from MS (13, 14, 39). The studies had adopted various methods, including multimodal data fusion, random forest classification, and hierarchical multimodal fusion, and achieved accuracies ranging from 74 to 88%. However, the applicability of the developed methods in clinical practice was uncertain owing to the lack of their comparison to assessments by clinicians. In our study, we demonstrated that the proposed model exhibits a performance comparable to that of trained clinicians, demonstrating its potential clinical applicability. Recent systematic reviews of studies on the comparison between performances of artificial intelligence and clinicians have reported that the performance of artificial intelligence was comparable to that of clinicians (40, 41).

Several limitations should be noted for the current study. Firstly, we conducted this study in a retrospective manner without external validation, which entails the risk of bias and lack of generalizability. Further, the study participants were of a single ethnicity (Korean), which implies that our result might not be applicable to patients from other ethnic backgrounds (42). Future prospective studies that incorporate data from other international centers and larger data samples can overcome this limitation. Secondly, spinal MRI and CSF findings, other important diagnostic clues (7, 43), may strengthen the performance of the deep learning-based model. In the present study, we have tried to develop a model based on minimal information, but we may add these variables when developing a model in the future. Lastly, the proposed deep learning-based model was trained for binary classification. Hence, this model is not an automatic brain MRI interpreter. Our results provide evidence that deep learning can support the objective differential diagnosis of MS and NMOSD. In the future, we may attempt to include other CNS diseases using this deep learning-based model to solve multiclass classification problems.

In conclusion, the proposed deep learning-based model was verified to be capable of differentiating NMOSD from MS with an accuracy comparable to that of neurologists, exhibiting distinct advantages in terms of the consistency of classification. The proposed model has the potential to aid differential diagnosis of two important CNS inflammatory diseases in clinical practice. Further research is necessary to determine the applicability of this model in clinical settings and to determine whether the utilization of the model can lead to improved patient care and prognoses.

## Data Availability Statement

The raw data supporting the conclusions of this article will be made available by the authors, without undue reservation.

## Ethics Statement

The studies involving human participants were reviewed and approved by Institutional Review Board of the Asan Medical Center. Written informed consent for participation was not required for this study in accordance with the national legislation and the institutional requirements.

## Author Contributions

D-WK and K-KK contributed to conception and design of the study. HK and YL wrote the manuscript. Y-ML, E-JL, and K-KK collected clinical and MRI data. YO and HWK classified the MRI data. HK, YL, and JL conducted data analysis. D-WK and E-JL acquired funding. Y-ML, E-JL, Y-HK, JW, S-KJ, D-WK, and K-KK supervised the study and revised the manuscript. All authors contributed to manuscript revision, read, and approved the submitted version.

## Conflict of Interest

The authors declare that the research was conducted in the absence of any commercial or financial relationships that could be construed as a potential conflict of interest.
